# Intravenous lidocaine infusion as a component of multimodal analgesia for colorectal surgery—measurement of plasma levels

**DOI:** 10.1186/s13741-019-0112-4

**Published:** 2019-02-26

**Authors:** E. Greenwood, S. Nimmo, H. Paterson, N. Homer, I. Foo

**Affiliations:** 1 Western General Hospital, Edinburgh, UK; 2 Queen’s Medical Research Institute, Edinburgh, UK

**Keywords:** Intravenous lidocaine infusion, Colorectal surgery, Plasma lidocaine concentration

## Abstract

**Background:**

Growing evidence suggests that intravenous lidocaine as a component of multimodal analgesia improves recovery after major colorectal surgery. There is little published data regarding ideal dosing and target plasma concentration in this context, and we wanted to establish our dosing schedule was safe by measuring blood levels of lidocaine.

**Methods:**

We measured the plasma lidocaine concentration of 32 patients at 30 min, 6 h and 12 h after starting intravenous lidocaine infusion for analgesia after major colorectal surgery. Patients received a bolus of 1.5 mg kg^−1^ over 20 min at the time of induction of anaesthesia. This was followed by a continuous infusion of 2% *w*/*v* lidocaine at 3 ml hr^−1^ (60 mg hr^−1^) for patients weighing up to 70 kg and 6 ml hr^−1^ (120 mg hr^−1^) for patients weighing over 70 kg, using actual body weight.

**Results:**

The overall mean plasma lidocaine concentration was 4.0 μg ml^−1^ (range 0.6–12.3 *μg* ml^−1^). In patients treated with the higher infusion dose, the mean concentration was 4.6 μg ml^−1^ compared to 3.2 *μg* ml^−1^ in those patients on the lower dose. Mean levels were higher at 6 h than 30 min and higher again at 12 h. There were no adverse events or reports of symptoms of local anaesthetic toxicity.

**Conclusions:**

Whilst there were no signs or symptoms of lidocaine toxicity in our patients, there was a wide range of plasma concentrations including some over 10 *μg* ml^−1^; a level above which symptoms of toxicity may be expected. We have changed our dosing protocol to using ideal rather than actual body weight based on these results.

## Background

Effective and safe acute pain management following major colorectal surgery is a challenge. Opioid drugs continue to make up the major component of multimodal analgesia, but are associated with significant side effects—in particular, contributing to postoperative ileus. The search for safe and effective alternatives and or adjuncts continues, and there has been a recent resurgence of interest in the use of intravenous lidocaine infusions. Studies to date have shown most success in patients undergoing major colorectal surgery, which may be due to both improved pain control but also reduction of ileus (Weibel et al. 2018; Ventham et al. 2015; Eipe et al. 2016; Rimback et al. 1990). However, questions remain regarding the details of mode of action and the optimal dosing schedule.

**Fig. 1:**
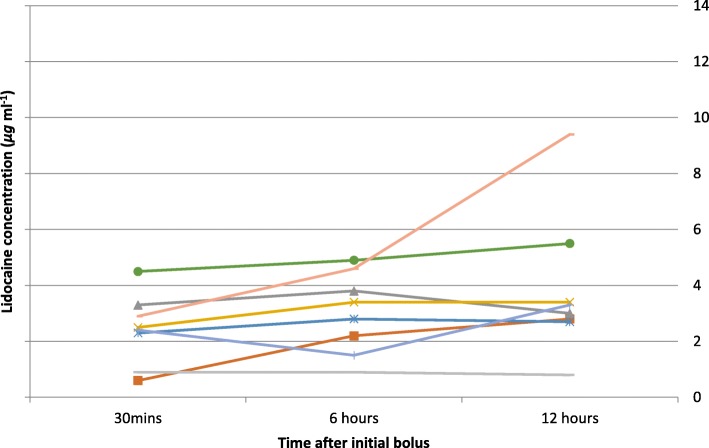
Plasma lidocaine levels in patients on the 60 mg hr^−1^ protocol in whom all three levels were obtained (8 patients)

**Fig. 2:**
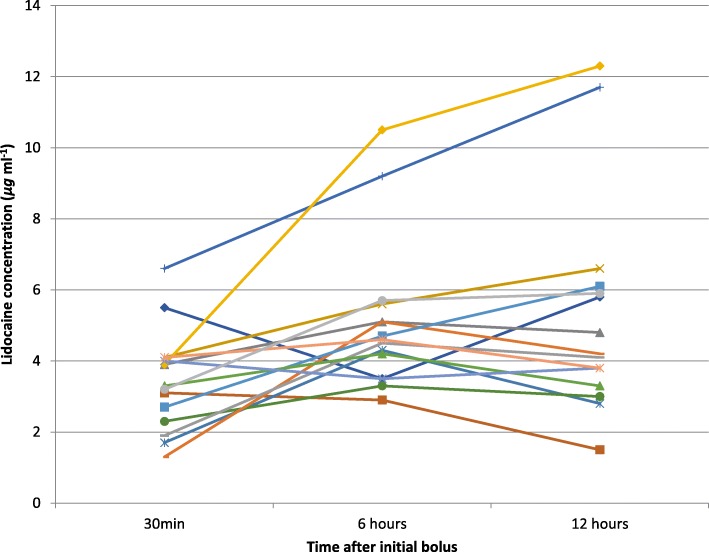
Plasma lidocaine levels in patients on the 120 mg hr^−1^ protocol in whom all three levels were obtained (15 patients)

The colorectal unit at the Western General Hospital in Edinburgh undertakes around 500 elective colorectal resections per annum utilising an enhanced recovery protocol (Tan et al. 2015; Fearon et al. 2005). We have collected audit data on over 2200 uses of intravenous lidocaine in these patients, with observed benefits in pain relief and ileus reduction as in other units. We have no incidence of local anaesthetic toxic side effects in any of these patients. Similarly, toxicity has been rare in other large published series (Weibel et al. 2018; Ventham et al. 2015). However, there is little recent work equating this with perceived safe plasma concentrations of lidocaine. Prior to embarking on a more formal study of the benefits of intravenous lidocaine, we undertook measurement of plasma lidocaine levels in a series of patients to assess whether our dosing schedule provided concentrations within a therapeutic and non-toxic range as defined by Foldes and Bromage (Bromage 1961; Foldes et al. 1960). The results are presented here (Figs. 1 and 2) (Table 1).

**Table 1:** Mean lidocaine levels with ranges (μg ml^−1^)

Time (hours)	60 mg hr^−1^	120 mg hr^−1^	All patients
0.5	2.3 (0.6–4.5)	3.3 (1.3–6.6)	2.8
6	3.0 (0.9–4.9)	5.1 (2.9–10.5)	4.2
12	4.2 (0.8–9.4)	5.3 (1.5–12.3)	4.9
All times	3.2	4.6	*4.0*

## Methods

All adult patients undergoing major colorectal resection in whom the surgical and anaesthetic teams planned to use intravenous lidocaine, as per local protocol, between March 2016 and May 2016 were considered for inclusion.

Advice was sought from our local ethics service regarding the requirement for ethical approval for this project, and since intravenous lidocaine is a routine part of care for these patients in our unit, this was considered to be a safety issue with no requirement for formal ethical committee review. Verbal consent was obtained from patients to take blood from arterial lines, as the assay required arterial blood samples. Patients in whom arterial lines were not deemed necessary were therefore not included in the study.

Patients treated with intravenous lidocaine received a bolus of 1.5 mg kg^−1^ over 20 min given at the time of induction of anaesthesia. This was followed by a continuous infusion of 2% *w*/*v* lidocaine at 3 ml hr^−1^ (60 mg hr^−1^) for patients weighing up to 70 kg and 6 ml hr^−1^ (120 mg hr^−1^) for patients weighing over 70 kg, using actual, rather than ideal body weight. Patients had continuous ECG monitoring throughout the duration of the infusion and staff were educated in monitoring for the early signs and symptoms of local anaesthetic toxicity. Absolute contraindications to intravenous lidocaine were known or suspected allergy to lidocaine or amide-type local anaesthetics, hypovolaemia and complete heart block. Infusion was through a dedicated intravenous cannula and a Y-connector/anti-syphon/anti-reflux line using an Omnifuse lockable syringe driver, pre-programmed with the infusion protocol. Intravenous sodium chloride 0.9% was infused at a rate of 10 ml hr^−1^ via the Y-connector.

A total of 85 arterial blood samples were collected and analysed. Samples were taken at 30 min, 6 h and 12 h after completion of the initial lidocaine bolus.

There were 20 female and 12 male patients. Eleven patients were on the lower dose protocol compared to 19 on the higher dose regimen. Two patients had missing information on samples so could not be included in the analysis, and some patients did not have samples taken at all three time intervals. The mean actual body weight of patients on the lower dose protocol was 66 kg (range 54–112 kg) and 91 kg (range 70–150 kg) on the higher dose protocol.

Samples were labelled, centrifuged, frozen and stored locally at − 80°. They were then sent as a single batch for analysis of total lidocaine concentration at the Queen’s Medical Research Institute, Edinburgh.

Lidocaine was extracted from plasma using an SLE+ plate (Biotage, UK) following enrichment with 3-nitrolidocaine as an internal standard. Calibration standards ranged from 1 to 2000 ng. Analytes were eluted, reduced to dryness under nitrogen and reconstituted in water/acetonitrile. Analysis was carried out by liquid chromatography-tandem mass spectrometry. Linear regression analysis of calibration standards, calculated using peak area ratios of lidocaine and 3-nitrolidocaine, was used to determine the concentration of lidocaine in the samples.

## Results

The overall mean plasma lidocaine concentration was 4.0 μg ml^−1^ (range 0.6–12.3 *μg* ml^−1^). There were no adverse events or reports of symptoms of local anaesthetic toxicity from any patients at any time points.

## Discussion and conclusion

The uses of lidocaine beyond local and regional tissue anaesthesia are well established. Given intravenously, it is effective in obtunding the sympathetic response to laryngoscopy, in reducing the pain of propofol injection and it is a class 1b antiarrhythmic agent. Some centres have demonstrated successful treatment of chronic neuropathic pain using intravenous lidocaine (Challapalli et al. 2009). Recent studies are establishing the benefits of postoperative lidocaine infusion, in particular after major gastrointestinal surgery as a component of multimodal analgesia (Weibel et al. 2018; Ventham et al. 2015). Benefits include analgesia, improvement in postoperative gut function and a feeling of euphoria (Weibel et al. 2018; Ventham et al. 2015; Koppert et al. 2004). The exact mechanism of action of lidocaine in this context is not yet fully understood, but it is known to have direct analgesic activity, to reduce central sensitisation to pain and has anti-inflammatory properties (Lauretti 2008).

Patient safety is of paramount importance, and although no patients had any symptoms suggestive of local anaesthetic toxicity in our series, and serious adverse events are extremely rare in the published meta-analyses, before progressing the use of intravenous lidocaine further, we wanted to undertake a series of plasma measurements to reassure ourselves of the safety of our technique. This has not been done before in this context and there is little published evidence available to guide plasma concentration targets. Measuring levels is not routinely offered by hospital laboratories in the UK; however, a very small number of papers describe monitoring levels acutely and adjusting treatment based on results (Weibel et al. 2018; Swenson et al. 2010).

Currently quoted therapeutic and toxic levels for plasma lidocaine are largely based on work from 1960 by Foldes and colleagues who gave 12 healthy volunteers rapid intravenous lidocaine boluses and measured levels (Foldes et al. 1960). Reported signs and symptoms included convulsions in two volunteers. A year later, Bromage published a study measuring levels in seven patients and correlating them to signs of toxicity (Bromage 1961). These studies suggested plasma concentrations above 5 μg ml^−1^ were associated with neurological symptoms and levels above 10 *μ*g ml^−1^ with cardiovascular instability. These papers are still widely referenced and appear to form the basis upon what is considered the ‘safe’ plasma concentration. Before this, in 1954, a British paper was published describing a case series of 1000 patients and administration of up to 750 mg per hour of intravenous lidocaine. There were three seizures reported, two of which were blamed on ‘administration error’ rather than complications of the intended treatment. Plasma levels were not measured. The authors concluded that their technique was safe and effective for intraoperative analgesia (Clive-Lowe et al. 1954). In comparison, the loading doses and infusion rates in our protocol are relatively conservative and are based on a studies such as the one by Swenson and colleagues (Swenson et al. 2010) who used 2 mg min^−1^ in patients weighing greater than 70 kg and 1 mg min^−1^ in patients less than 70 kg. It is interesting to note that they changed their regimen during their study after finding that on their original protocol (using 3 mg min^−1^ in patients > 70 kg and 2 mg min^−1^ in patients < 70 kg), several patients reached potentially toxic plasma levels (although not all absolute levels are reported in the paper), including one of their 22 study patients who experienced disorientation and hallucinations after 4 days of intravenous lidocaine. This patient was reported to have had a plasma lidocaine concentration of 6.5 μg ml^−1^ at the time. It is worth considering that the clinical effects noted in this case could have been due to the accumulation of lidocaine metabolites as nonlinear pharmacokinetics is demonstrable in extended lidocaine infusions (LeLorier et al. 1977; Weinberg et al. 2015). Our protocol runs for only 12 h so it avoids this potential problem.

Peak plasma concentrations and importantly, clinical evidence of toxicity, is related to total dose, but also rate and duration of infusion. A rapid bolus is more likely to lead to neurological symptoms (Bromage 1961). In publications describing intravenous lidocaine infusions as a component of multimodal analgesia for perioperative pain, there is an assumption that plasma levels are below the toxic concentrations described by Foldes and Bromage. Our results, showing some patients to have lidocaine levels well above the threshold for toxicity, are therefore important for a number of reasons. Firstly, these levels could be associated with both neurological and cardiovascular toxicity highlighting the importance of appropriate monitoring and staff training in the management of toxicity including the use of intralipid. Secondly, since neither we nor other clinicians routinely using intravenous lidocaine have seen any symptoms of toxicity, even mild ones, it is pertinent to ask whether we need to revise the quoted levels in the context of much slower administration rates than those used in the original trials investigating toxic doses. All patients were under general anaesthesia at the time the first sample was taken, at 30 min; therefore, symptoms of toxicity could of course not be assessed. However, we found the highest levels were at 12 h, and therefore, patients were fully recovered and able to report symptoms of toxicity, had they occurred. Additionally, since the optimal infusion rate and duration are not yet known in the context of postoperative pain management, future work should include measurement of plasma levels as well as monitoring for signs and symptoms of toxicity. On review, the patients with the highest levels in our study were emergency cases, who in retrospect may have had organ impairment and hypovolaemia contributing to altered lidocaine metabolism and higher plasma concentrations (deOliveira et al. 2010).

Our original protocol was designed using two fixed dosing schedules, as well as actual rather than ideal body weight for doses, in order to reduce the risk of administration error and make dosing calculations more straight forward. In view of finding that heavier patients on the 120 mg hr^−1^ dose protocol had higher mean levels, including two patients with plasma concentrations greater than 10 μg ml^−1^, and to attempt to create consistency in plasma concentrations in our patients, we changed our protocol to use ideal rather than actual body weight for dosing. We propose future work relating to intravenous lidocaine should clearly state whether lean, ideal or actual body weight is used for dosing, as little published work to date specifies this.

The Western General Hospital is one of the largest colorectal units in the UK. In the last decade, there has been a major shift from open to laparoscopic surgery. This has necessitated a change in anaesthetic practice to facilitate enhanced recovery in this group of patients. Our clinical experience with intravenous lidocaine over 2200 patients to date has confirmed benefits in improved quality of recovery and improved return of gut function. This is particularly relevant, since the occurrence of ileus in up to one third of laparoscopic colorectal resection patients is the single commonest reason for prolonged recovery and hospital stay. These findings correlate with the meta-analyses (Weibel et al. 2018; Ventham et al. 2015). We feel this study makes an important contribution to the mounting evidence that intravenous lidocaine infusion after laparoscopic surgery is safe and effective.
